# Octopamine and Tyramine Contribute Separately to the Counter-Regulatory Response to Sugar Deficit in *Drosophila*

**DOI:** 10.3389/fnsys.2017.00100

**Published:** 2018-01-15

**Authors:** Christine Damrau, Naoko Toshima, Teiichi Tanimura, Björn Brembs, Julien Colomb

**Affiliations:** ^1^Neurobiologie, Fachbereich Biologie-Chemie-Pharmazie, Institut für Biologie - Neurobiologie, Freie Universität Berlin, Berlin, Germany; ^2^Division of Biological Sciences, Graduate School of Systems Life Sciences, Kyushu University, Fukuoka, Japan; ^3^Institute of Zoology – Neurogenetics, University of Regensburg, Regensburg, Germany

**Keywords:** biogenic amines, starvation, starvation resistance, insects, proboscis extension response

## Abstract

All animals constantly negotiate external with internal demands before and during action selection. Energy homeostasis is a major internal factor biasing action selection. For instance, in addition to physiologically regulating carbohydrate mobilization, starvation-induced sugar shortage also biases action selection toward food-seeking and food consumption behaviors (the counter-regulatory response). Biogenic amines are often involved when such widespread behavioral biases need to be orchestrated. In mammals, norepinephrine (noradrenalin) is involved in the counterregulatory response to starvation-induced drops in glucose levels. The invertebrate homolog of noradrenalin, octopamine (OA) and its precursor tyramine (TA) are neuromodulators operating in many different neuronal and physiological processes. Tyrosine-ß-hydroxylase (*tßh*) mutants are unable to convert TA into OA. We hypothesized that *tßh* mutant flies may be aberrant in some or all of the counter-regulatory responses to starvation and that techniques restoring gene function or amine signaling may elucidate potential mechanisms and sites of action. Corroborating our hypothesis, starved mutants show a reduced sugar response and their hemolymph sugar concentration is elevated compared to control flies. When starved, they survive longer. Temporally controlled rescue experiments revealed an action of the OA/TA-system during the sugar response, while spatially controlled rescue experiments suggest actions also outside of the nervous system. Additionally, the analysis of two OA- and four TA-receptor mutants suggests an involvement of both receptor types in the animals' physiological and neuronal response to starvation. These results complement the investigations in *Apis mellifera* described in our companion paper (Buckemüller et al., 2017).

## Introduction

There may be more than just cultural value to the old German saying “grain tastes bitter for a satiated mouse” (La Sala et al., 2013). Indeed, it is the state of an organism which determines what, if any, effect external sensory stimuli will have on the nervous system. Whether this is the satiation state of the mouse influencing taste receptors, or the feeding state of the leech which gates mechanosensory stimuli (Gaudry and Kristan, 2009, 2010), or the locomotor state of flies which adjusts the gain in visual interneurons (Longden and Krapp, 2009; Chiappe et al., 2010; Maimon et al., 2010; Suver et al., 2012; Tuthill et al., 2014; van Breugel et al., 2014), sensory stimuli are rarely, if ever, directly transformed into motor outputs. Instead, all nervous systems seem to constantly balance external and internal demands before they arrive at any given action (Heisenberg, 2009; Brembs, 2013, 2017; Pezzulo and Cisek, 2016). Biogenic amines and neuropeptides have been shown to be crucially involved in orchestrating the processes needed to find this balance.

Starvation and satiation are obvious and experimentally accessible states with immediate and easily recorded behavioral consequences. In both mammals and insects, peptides (glucagon and adipokinetic hormone, respectively) and catecholamines (adrenaline and octopamine, respectively) have been shown to mediate related roles in the counterregulatory response to starvation (Bolli and Fanelli, 1999; Kim and Rulifson, 2004; Grönke et al., 2007; Bharucha et al., 2008; Li et al., 2016; Yu et al., 2016). Apparently, either similar mechanisms evolved in response to similar challenges, or both systems evolved from a common ancestor. This response includes various physiological and metabolic modifications, which are orchestrated via the different neuropeptides and biogenic amines.

Feeding-related behaviors constitute the behavioral aspect of the counterregulatory response to starvation. In flies, general activity and arousal is enhanced (Connolly, 1966; Bell et al., 1985; Lee, 2004; Yang et al., 2015; Yu et al., 2016), arguably to facilitate food discovery. Along the same veins, food sensitivity is also increased (Moss and Dethier, 1983; Colomb et al., 2009), correlated with an increase in sugar receptor neuron sensitivity and gene expression (Amakawa, 2001; Meunier et al., 2007; Nishimura et al., 2012). Several involved neuropeptides have been identified (for a review see: Nässel and Winther, 2010). In addition to neuropeptides, also here the catecholamines are contributing to the processes triggered by starvation. Dopamine (DA) is involved in mediating motivation signals (Krashes et al., 2009) and modulating the starvation-induced sugar response after short starvation periods (Inagaki et al., 2012), while octopamine (OA) or its precursor tyramine (TA) have been reported to promote feeding behaviors (Long and Murdock, 1983; Nisimura, 2005). Starvation may be conceived as a stressor triggering catecholaminergic action. Indeed, different stressors have been shown in different insects to modify the OA/TA-system by enhancing Tßh expression (Châtel et al., 2012), subsequently increasing OA levels (Kononenko et al., 2009), which, in turn, releases triglycerides and carbohydrates into the hemolymph (Woodring et al., 1989).

The study of the role of biogenic amines in the counterregulatory response to starvation is complicated by the amines' broad involvement in many physiological processes. This promiscuity impedes the attribution of an aminergic manipulation to a specific phenotype. In invertebrates, OA and TA act as neurotransmitters, -hormones, and -modulators on many, if not all, physiological processes (reviews: Roeder, 2005; Farooqui, 2012). These processes include, but are not limited to, locomotion regulation (Saraswati et al., 2004; Brembs et al., 2007), aggression (Baier et al., 2002; Hoyer et al., 2008; Zhou et al., 2008), reaction to light (Gorostiza et al., 2016), feeding behavior (Long and Murdock, 1983; Nisimura, 2005), mobilization of energy metabolites (Mentel et al., 2003) and, upstream of DA, appetitive olfactory learning (Hammer, 1993; Schwaerzel et al., 2003; Burke et al., 2012; Liu et al., 2012).

Thus, while feeding behaviors and their interactions with the state of the animal provide a technically accessible model to study decision-making and action selection, the interrelation between the consequences of starvation on motor control, motivation, stress, and the metabolic state of the animal pose a formidable experimental challenge, in particular in the interpretation of the different phenotypes linked with biogenic amine disruption. Leveraging the neurogenetic tools in *Drosophila*, we attempted to understand how starvation influences the animal's decision-making with regard to feeding-related stimuli. Specifically, we investigated the involvement of the OA/TA-system on starvation-dependent modulation of sugar responsiveness and metabolism. We asked whether the OA/TA-system was involved in the physiological response to starvation or the neuronal changes following starvation, and whether its neuronal action was peripheral or central. Our results corroborate and extend the previous findings on the promiscuous effects of these biogenic amines and suggest that both OA and TA are involved in most of the counterregulatory processes, which occur in parallel.

## Methods

### External depositories

A formatted table of most reagents used in this study, including fly stocks, is available at: https://doi.org/10.6084/m9.figshare.5398600. The data and code for this paper are available at https://doi.org/10.6084/m9.figshare.4663666. Protocol for carbohydrate measurement is available on protocols.io: https://doi.org/10.17504/protocols.io.dkn4vd.

### Fly stocks and culture

*tßh*^*nM18*^ (Monastirioti et al., 1996; FBal0061578), *oamb* (Han et al., 1996; *oamb*^*286*^ FBal0152344, *oamb*^*584*^ FBal0152335), *honoka* (Kutsukake et al., 2000; *Oct-TyrR*, FBal0104701), *hsp-tßh* (Schwaerzel et al., 2003; FBal0152162), and w+;;*UAS-tßh* (Monastirioti, 2003; FBti0038601) and their control lines were obtained from Henrike Scholz, Cologne; Hiromu Tanimoto, Martinsried; Andreas Thum, Konstanz; and Amita Seghal, Chevy Chase. *TyrR*^*f05682*^ (CG7431f05682, FBal0184987), *TyrRII*^*Δ29*^ (CG16766, FBgn0038541) and *TyrRII-TyrR*^*Δ124*^ were kindly provided before publication by Edward Blumenthal, Milwaukee (Zhang and Blumenthal, 2017). Receptor mutants (and the respective control lines we obtained from the different labs) were outcrossed for at least six generations into a CS background. Flies were kept on standard cornmeal/molasses-food in a 12/12 h light/dark cycle (light on at 8:00 h) at 60% relative humidity and 25°C except for *hsp-tßh*, which were raised at 18°C without humidity control and except for flies used in electrophysiological experiments (see Electrophysiological recordings).

### Starvation procedure

Newly hatched to 1-day-old flies were collected and transferred to fresh food vials. The following day (between 16:00 and 19:00 h), 20 to 30 flies of mixed sexes were transferred into starvation vials (68 ml, Greiner bio-one, Frickenhausen, Germany) by a fly aspirator. The starvation vial contained a cotton pad moistened with 2.5 to 3 ml of Evian® water. If not otherwise indicated, starvation was performed at 25°C and 60% relative humidity and lasted for 20 h. Note that starvation time at 18°C was performed for much longer time.

### Survival experiments

Newly hatched to 1 day-old flies were collected and transferred to fresh food vials. The following day, flies were briefly CO^2^-anesthetized and sorted by sex and genotype. At 17:00 h, around 35 female flies were transferred into a starvation vial (see Starvation procedure). Dead flies were counted every 3 h and not removed. Daily counting sessions were repeated from 9:00 to 18:00 h, until all flies were found dead.

### Sugar response test

Newly hatched to 1 day-old flies were collected and transferred to fresh food vials. The following day, they were starved as described (see Starvation procedure). Four hours before the end of the starvation period, female flies (if not stated otherwise) were briefly immobilized by cold-anesthesia. Their head and thorax were glued to a triangle-shaped copper hook (0.05 mm in diameter) using a UV sensitive glue (3M ESPE, Sinfony Indirect Lab Composite, Minneapolis, USA). Animals were then kept individually in small chambers [14 mm in diameter × 28 mm in height, custom-made, (Brembs, 2008)] with *ad libitum* access to water until the test.

Tests were performed between 12:00 and 16:00 h. Using forceps, we transferred flies by their hook and fixed them to a magnetic clamp, which was then attached to a rack. This treatment established free movement of the flies' tarsi and proboscis and was a modication from a previously described PER assay (Scheiner et al., 2004, 2014) derived from assays used in other insects (Dethier, 1952; Page et al., 1998). A group of six to eight flies was tested in parallel. A filter paper soaked with sucrose solution was presented for 5 s to all six tarsi but not the proboscis. Seven different, increasing concentrations (0, 0.1, 0.3, 0.6, 1, 3, and 30%, i.e., g per 100 ml water) were presented in series with an inter-stimulus interval of 80 s. The proboscis extension response was recorded. Finally, the proboscis was stimulated by 30% sucrose solution. Flies not responding to the proboscis stimulation or responding to the first stimulation (water only) were discarded from the analysis.

For the first sugar response rescue attempt (**Figure 4A**), flies were raised and starved at 18°C and put into an incubator without humidity control and heated up to 37°C for 30 to 45 min. After the heat shock, flies were kept in a 25°C incubator with humidity control for 3 h until testing. For the second rescue attempt (**Figure 4B**), the first heat shock was given with the beginning of starvation every 23 h for 45 min until 1 day before testing. Temperature between heat shocks was 18°C.

### Carbohydrate measurement

Newly hatched to 1 day-old flies were collected and transferred to fresh food vials. The following day at 17:00 h, 20 flies of mixed sex were either transferred into starvation vials (see Starvation procedure) or kept in the food vials. After 20 h, approximately 40 female flies per group were cold-anesthetized, pierced through the thorax by the tip of a dissecting needle (0.5 mm in diameter), and collected on ice within a sieve composed of two tubes. The hemolymph was centrifuged out of the fly into the bottom tube at 4°C. 0.5 μl of the extracted hemolymph was transferred by a capillary (0.5 μl, Hirschmann Laborgeraete, Eberstadt, Germany) into 19.5 μl PBS (see https://doi.org/10.17504/protocols.io.dkn4vd).

Trehalose and glucose content in the hemolymph were measured according to the protocols provided by the manufacturer (Sigma Aldrich, Seelze, Germany). Ten microliter of the hemolymph-PBS mixture (or calibration solution) were added to 30 μl citric acid buffer (135 mM, pH 5.7 at 37°C) and 10 μl of a trehalase enzyme solution (Sigma Aldrich, 3% in citric acid buffer). After incubation overnight at 37°C, 50 μl of Tris buffer were added. 80 μl of the resulting solution were added to 156.8 μl Glucose oxidase and 3.2 μl o-Dianisidine (Glucose Assay Kit, Sigma Aldrich) and incubated for 30 min at 37°C. Finally, 160 μl of 33% sulfuric acid were added. Absorbance at 540 nm was measured for the resulting solution using a nanoDrop® (nanoDrop Technologies, Wilmington, USA) spectrophotometer. Five samples were measured per solution.

### Electrophysiological recordings

Flies were raised on cornmeal-yeast-glucose-agar medium under a 12/12 h light/dark cycle (lights on at 06:00 h) at 25°C. Newly hatched to 1 day old flies were collected and transferred into a vial containing Kimwipe paper soaked with 100 mM glucose for 1 to 2 days as previously described (Zhang et al., 2010). Starved flies were kept in a vial containing Kimwipe paper soaked with Evian® water for 20 h before testing.

Electrophysiological recordings from l-type labellar chemosensilla were done by the tip-recording method, as previously described (Hodgson et al., 1955; Hiroi et al., 2002). Briefly, the proboscis was fixed at the base of the labellum. A glass capillary filled with Drosophila Ringer solution served as an indifferent electrode. The 100 mM sucrose solution for stimulation contained 1 mM KCl as electrolyte. The recorded signals were digitized and analyzed using the custom software dbWave (Marion-Poll, 1995, 1996). Action potentials were detected by a visually-adjusted threshold set across the digitally filtered signal. The total number of spikes within 1 s was counted. Note that in the tip-recording assay, recording and stimulation of the sensory neurons starts concomitantly.

### Statistics

Figures and statistical analyses were performed in R using different packages (Venables and Ripley, 2002; R Core Team, 2015; Therneau, 2015; Wickham, 2016; Wilke, 2016); data and code are available https://doi.org/10.6084/m9.figshare.4663666. If not stated otherwise, data are illustrated as boxplots representing the median (line), the 25 and 75% quartiles (boxes), the data within 1.5 times the interquartile range (whiskers), and data outside that range (outliers, depicted as points). Colors were chosen to be color-blind friendly, according to http://jfly.iam.u-tokyo.ac.jp/color/.

The sugar response score was calculated as the sum of all positive responses over the seven sucrose presentations and therefore ranges from 0 to 7 (Total number of PER). For survival measurement, we used Kaplan-Meier curves and Cox proportional hazards regression model. For hemolymph carbohydrate content, we used a paired Wilcoxon rank sum test on the index of change in sugar content with starvation: (SG_starved − SG_fed)/(SG_starved + SG_fed). Since one calibration experiment (showing the absorbance of a standard glucose/trehalose solution that was treated identically to hemolymph) was performed each day, a paired test is sound. In the 2 days with two measures per group, values were paired following time of measurement.

The significance level of all statistical tests was set to 0.05, and Bonferroni correction was applied where appropriate.

## Results

### *tβh*^*nM18*^ mutants in starvation induced phenotypes

We developed a new sugar response assay independent of the flies' locomotion, and which restrains their movements to less than in a pipet tip (Scheiner et al., 2004). Flies were tethered to a hook glued between head and thorax and tested for their proboscis extension response to a serial dilution of sucrose after 20 h of starvation. The assay is quite sensitive, since we were able to record a difference in the response of flies starved for 14 vs. 21 h (Damrau et al., 2014), as in the T-maze assay (Colomb et al., 2009). Fed flies do not respond to tarsal stimulation, in contrast to honeybees.

**Figure 1:**
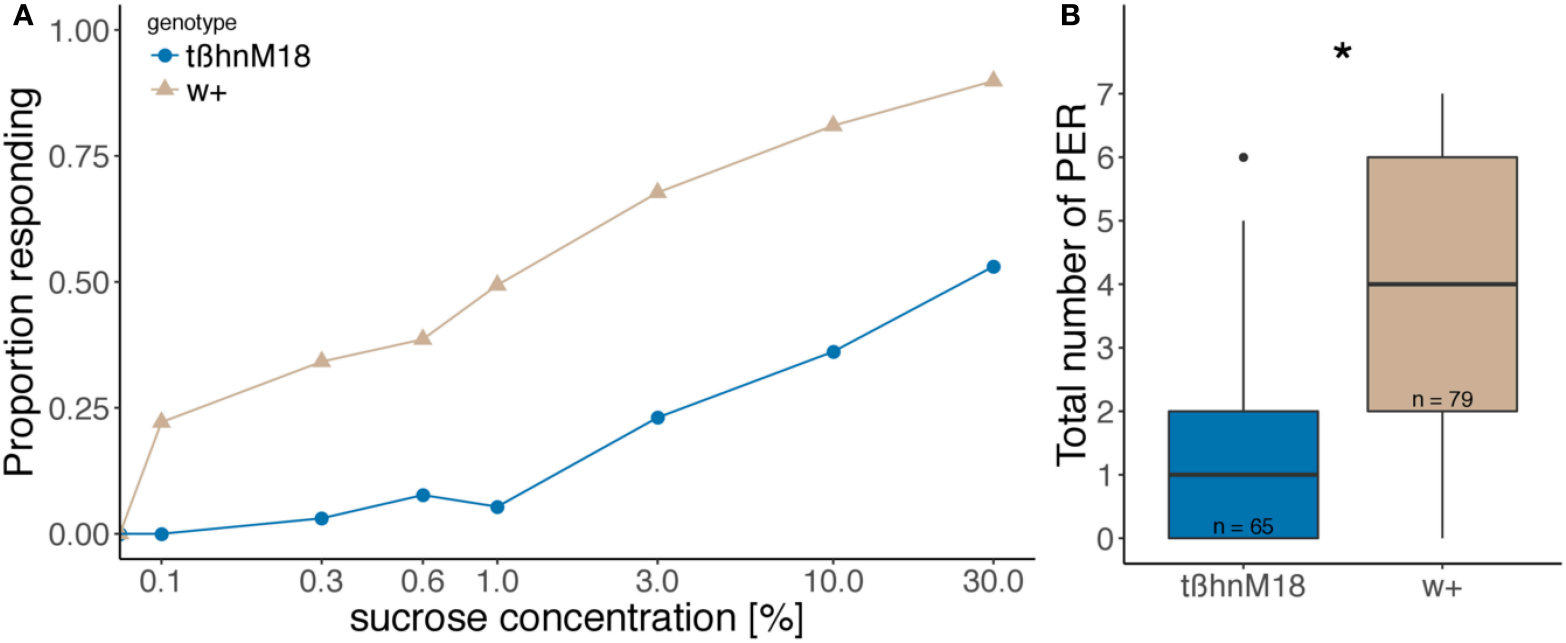
Reduced sugar response in *tßh*^*nM18*^ mutant flies compared to wild-type flies after 20 h of starvation. **(A)** Fraction of flies that responded to each concentration of sucrose. **(B)** Total number of positive responses. Boxplots represent the median (bar), the 75- and 25%-quartiles (box) and data within 1.5 times the interquartile range (whiskers). Data outside 1.5 times the interquartile range are considered as outliers (black dots). Numbers indicate sample size, asterisk denotes significant difference between genotypes (Wilcoxon rank sum test, *p* = 1.8 × 10^−12^).

We tested females *tßh*^*nM18*^ mutant flies lacking OA and accumulating TA (Monastirioti et al., 1996) in our assay. *tßh*^*nM18*^ mutant flies responded almost 40% less than their control (called w+ because the mutant and the control lines have a wild-type white gene, in contrast to the original mutant obtained after P element excision, Figure 1A). The sum of all positive responses over the 7 sucrose presentations was significantly different (Figure 1B).

**Figure 2:**
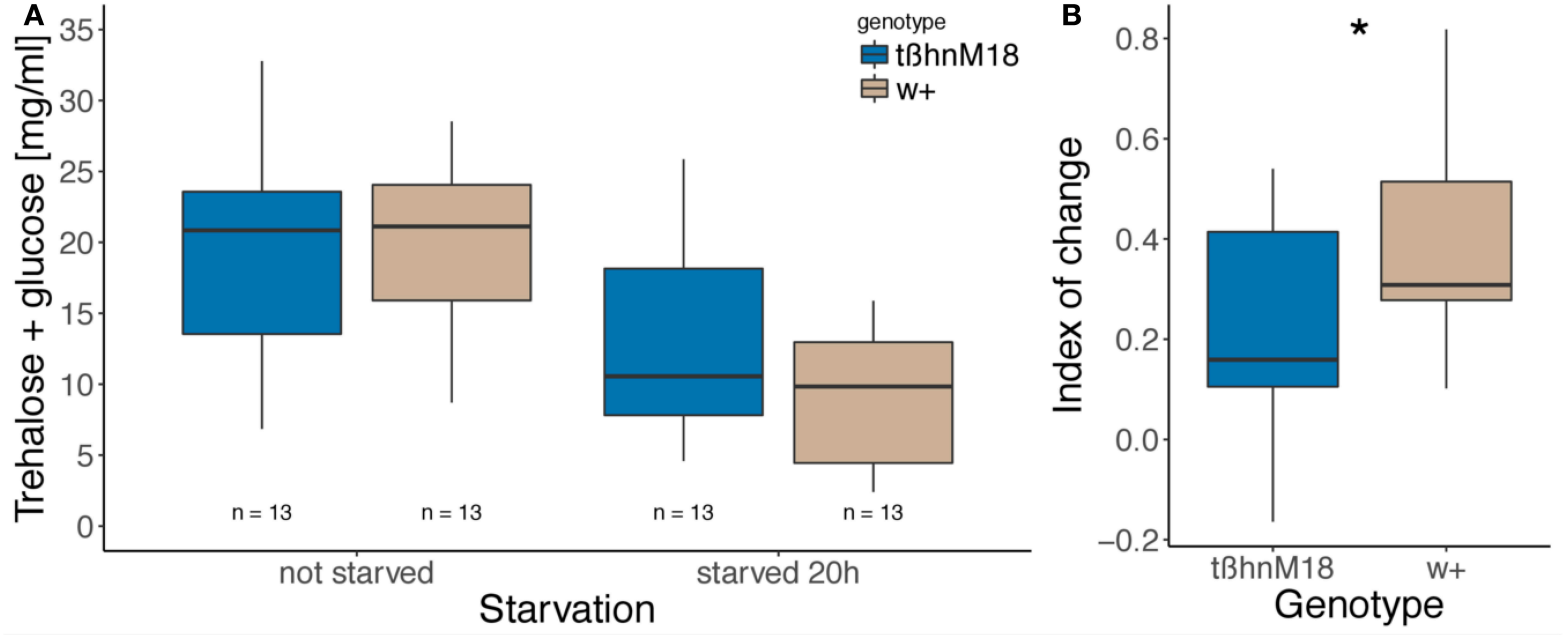
Change in hemolymph glucose and trehalose after starvation is smaller in *tßh*^*nM18*^ mutants than in wild type. **(A)** Concentration of trehalose and glucose in the hemolymph of fed and 20 h starved flies, which was calculated from absorbance at 540 nm compared to calibration solutions, is shown in boxplots. Numbers indicate sample size. No statistical test was applied. **(B)** Index of the change (difference over the sum of the two numbers) in absorbance between the starved and the fed fly, paired per day. Numbers indicate sample size, asterisk denotes significant difference between genotypes (paired Wilcoxon rank sum test, *p* = 0.03223).

We then compared the change in carbohydrate contents (trehalose plus glucose) in the hemolymph of starved and fed flies. To this end, the hemolymph was extracted and all glucose and trehalose was enzymatically converted into spectrometrically measurable glucose. Carbohydrate content in fed animals appeared very similar (Figure 2A, no statistics performed). Because the variability in the score is partly due to the inevitable differences in the manipulations from 1 day to another, we evaluated the change in carbohydrate level after starvation in a paired fashion. It was significantly smaller in *tßh*^*nM18*^ mutants compared to wild-type controls (Figure 2B).

**Figure 3:**
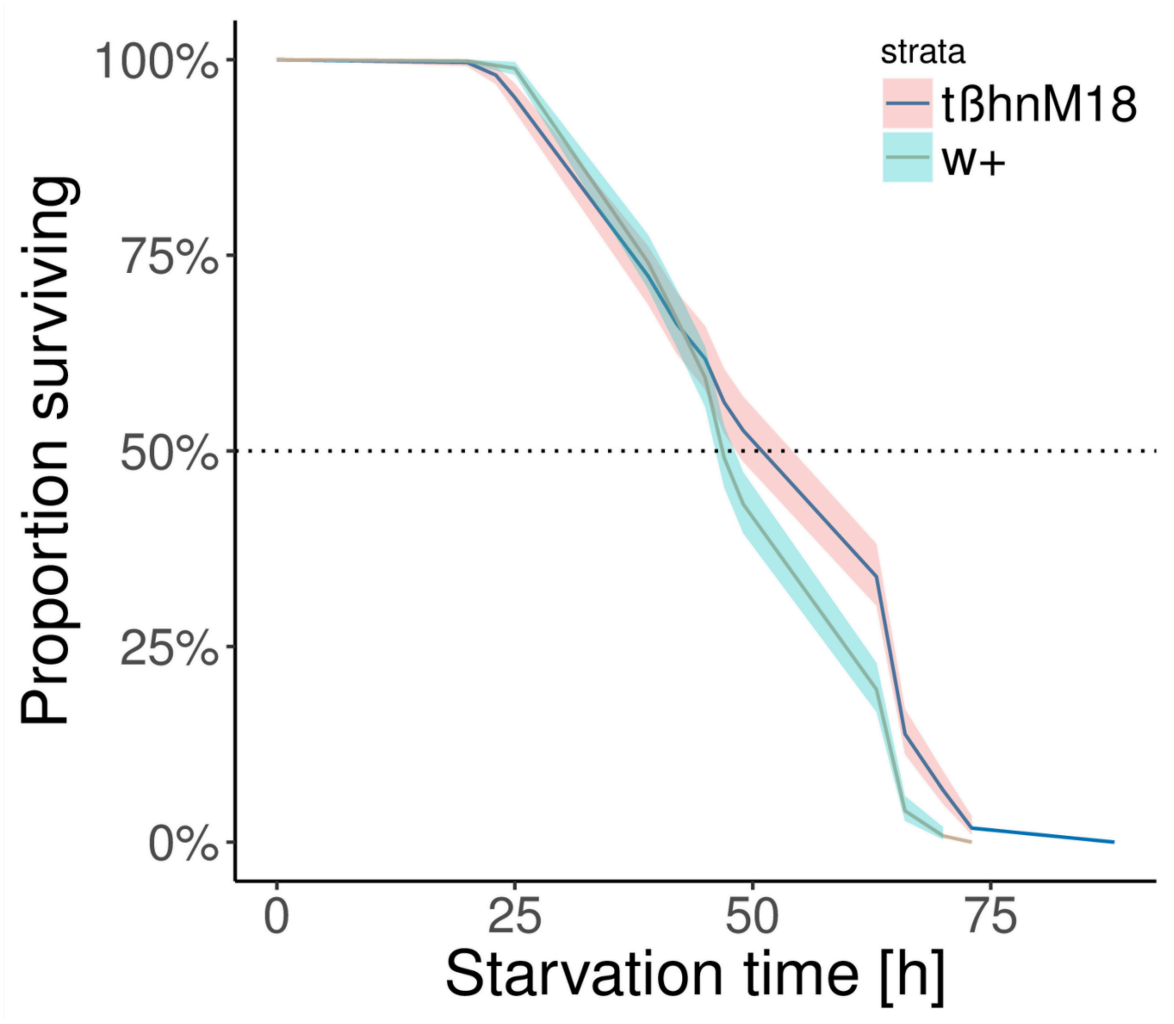
Longer survival of *tßh*^*nM18*^ mutants under starvation conditions. **(A)** Kaplan Meier survival curve for the two genotypes. We ran 16 experiments with about 35 flies per vial. The difference between the curves was statistically significant, while there was also an effect of the different trials (Cox proportional hazards regression model, *p* = 0.029 for trials, *p* = 2 × 10^−9^ for genotypes).

Finally, we recorded survival rate under starvation conditions with *ad libitum* access to water. As expected from the smaller decrease in their sugar content, *tßh*^*nM18*^ mutants survived longer than wild-type controls (Figure 3). Our experiments show that *tßh*^*nM18*^ mutants are less affected by starvation than wild-type animals, suggesting a role for OA and/or TA in starvation resistance and sugar response.

### OA/TA role in sugar response

**Figure 4:**
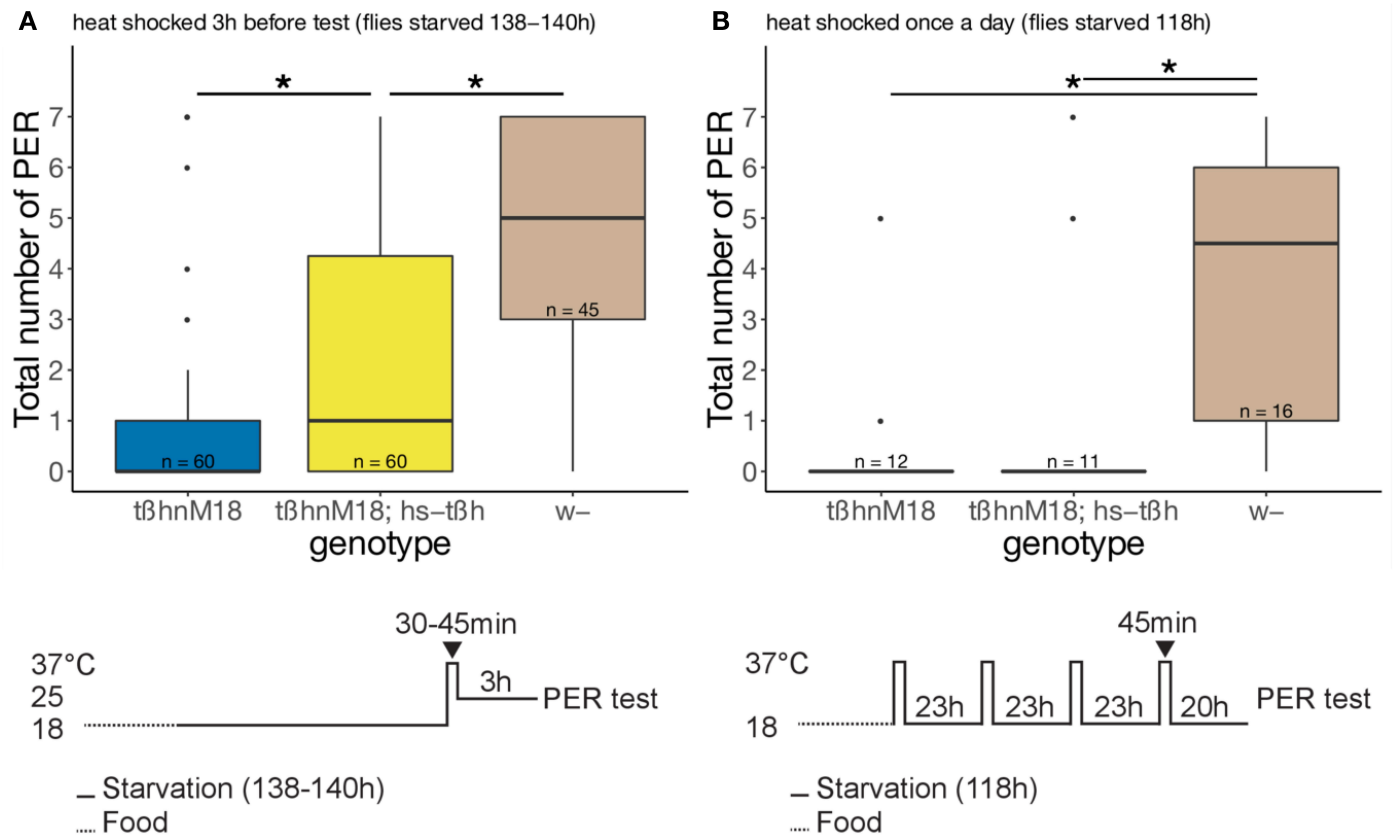
Effects of temporally controlled (ubiquitous) expression of Tßh in *tßh*^*nM18*^ mutant background on flies sugar response. **(A)** Temperature shift 3 h before the test. Flies with a rescue construct showed an intermediate PER level, significantly different from both the mutant and the control flies, Wilcoxon rank sum test with Bonferroni correction (uncorrected *p* = 0.00019). **(B)** Temperature shifts during the starvation period, but not immediately before the test. Flies with a rescue construct behaved similarly as mutant flies. Total number of proboscis extension responses is represented in boxplots (see Figure 1). Numbers indicate sample size, asterisks denote significant difference between groups (paired Wilcoxon rank sum test, *p* = 0.00041 and 0.00781).

In order to elucidate the temporal requirement of *tßh* activity during starvation or during proboscis extension, we induced ubiquitous, but temporally controlled, *tßh* expression in the mutant background using the heat-inducible *hsp-tßh* construct. To prevent *tßh* expression, flies were kept at 18°C, and the starvation time was increased to until the wild-type flies responded to sugar stimulation in a similar way as after 25°C starvation (see materials and Methods and Figure 1). Driving expression 3 h before the test partially rescued the mutant phenotype (Figure 4A). In contrast, heat shocks throughout the starvation period did not rescue the sugar response phenotype (Figure 4B), suggesting an acute role of OA during the sugar response test, independent of any OA/TA role in starvation resistance.

**Figure 5:**
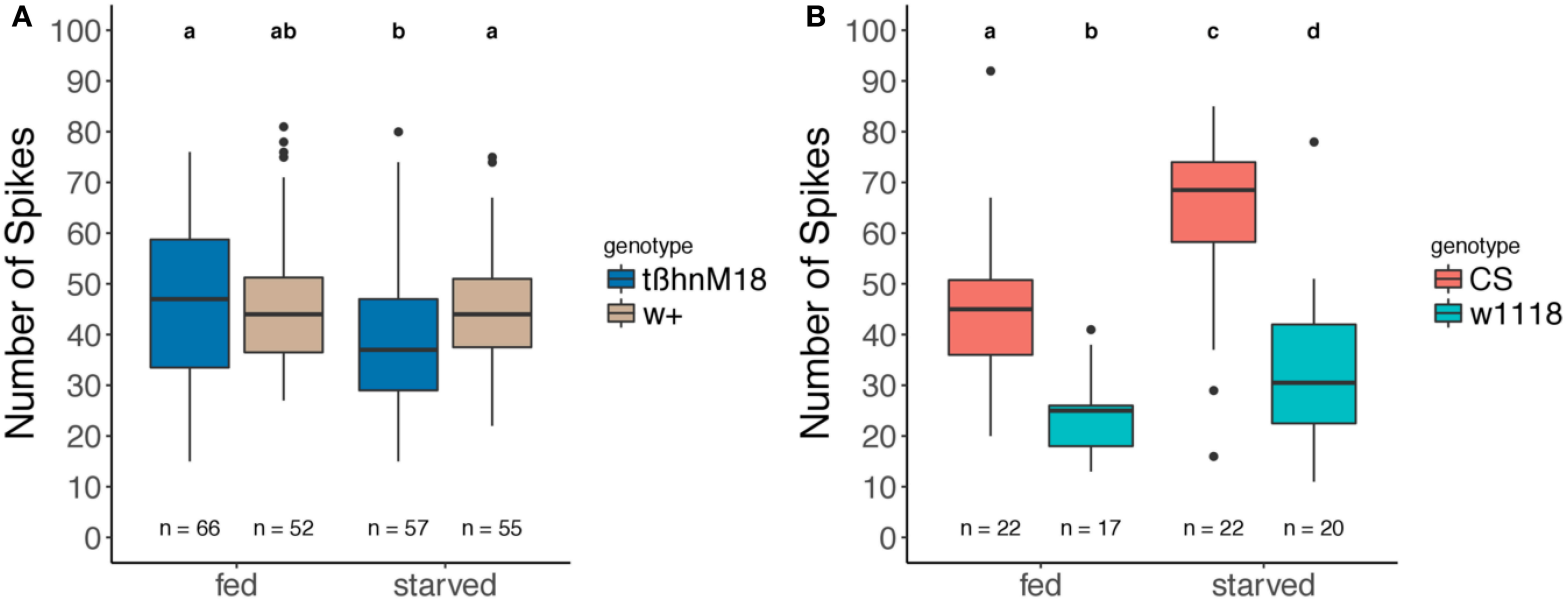
Effect of starvation on taste neuron sensitivity: electrophysiological recording from different gustatory sensilla on the labellum. **(A)** In sated and starved *tßh*^*nM18*^ mutants and their respective controls and **(B)** in sated and starved usual wild-type flies. Extracellular action potentials within 1 s after stimulation onset were counted and plotted as boxplots. Numbers represent the sample size of the recorded sensilla, Different letters denote significant differences (paired Wilcoxon rank sum test, **(A)**: *p* = 0.037 and 0.048, with Bonferroni correction, **(B)**: w1118 *p* = 0.03938, CS, *p* = 0.00174).

Since OA is known to modulate different kinds of sensory receptors in insects (Kass et al., 1988; Ramirez and Orchard, 1990; Pophof, 2000), we tested a potential role of OA on gustatory receptor sensitivity. We recorded the response of labellar sensilla to 100 mM sucrose in fed and starved flies by the tip-recording method (Hodgson et al., 1955; Hiroi et al., 2002). The wild-type strain serving as a control for our mutant does not show the increase of spiking rate after starvation (Figure 5A), which we see in other wild-type strains (Figure 5B) as previously reported (Meunier et al., 2007; Inagaki et al., 2012; Nishimura et al., 2012); and we found a decreased sensillar response to sucrose stimulation after starvation in *tßh*^*nM18*^ mutants, compared to starved wild-type controls and fed mutants (Figure 5A).

**Figure 6:**
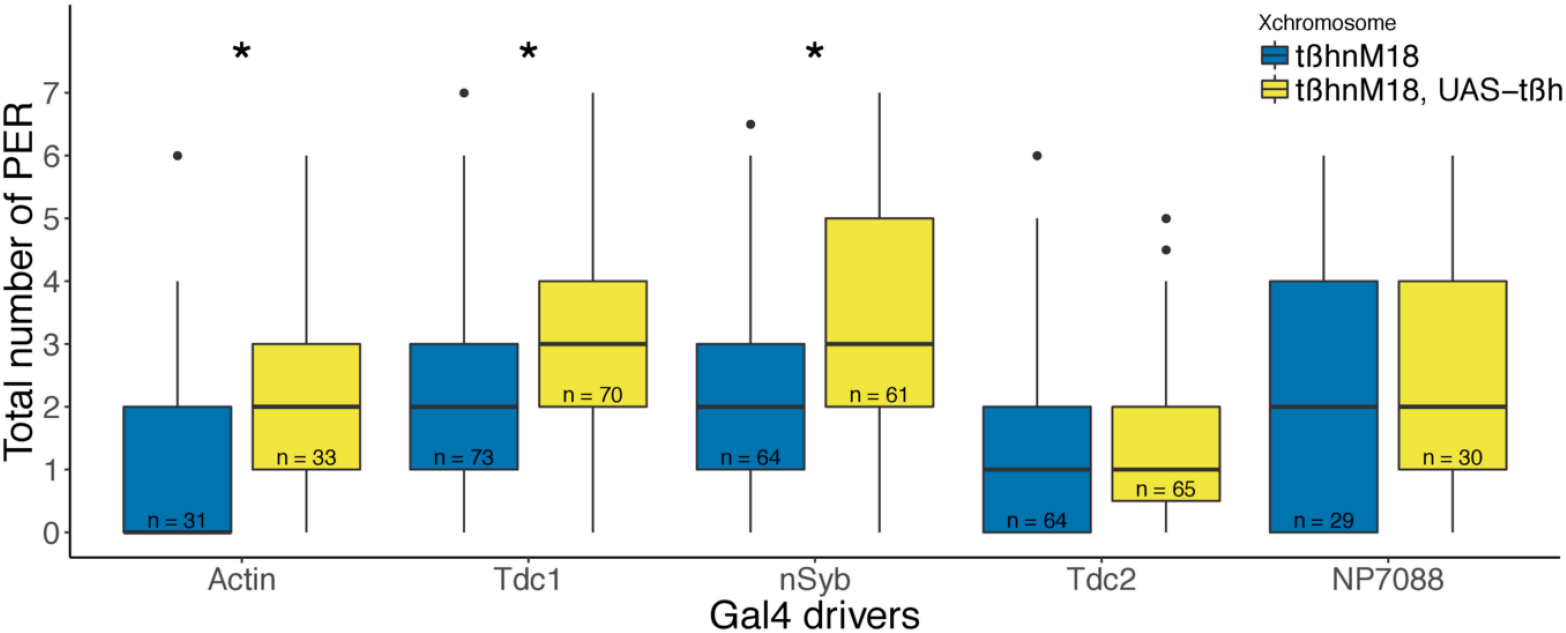
Spatially controlled Tßh expression in *tßh*^*nM18*^ mutant background. Ubiquitous (actin), pan-neuronal (nSyb) and non-neuronal TDC (Tdc1) drivers significantly increased sugar responsiveness. Neuronal TDC (tdc2) and OA (NP7088) specific drivers did not alter sugar responsiveness. Boxplots depict total number of proboscis extensions in hemizygous mutant males with or without a UAS-tßh construct, and heterozygous for the Gal4 driver. Numbers indicate sample sizes, asterisks denote significant difference between the mutant its respective rescue group (Wilcoxon rank sum test, Actin *p* = 0.01621, Tdc1 *p* = 0.02782, nSyb *p* = 0.01341).

OA and TA can act both inside and outside of the nervous system, functioning as either a neurotransmitter or a neurohormone in insects (Cole et al., 2005). Thus, we explored whether the sugar response phenotype of *tßh* mutants was a result of alterations in neurons inside or outside of the brain or in non-neuronal cells. To this end, we expressed Tßh in *tßh*^*nM18*^ mutant males using different GAL4-lines. We found a significant increase in sugar response compared to the respective mutant control when we used the ubiquitous Actin-promoter to drive Gal4 in all cells, the pan-neuronal nSyb-promoter, or the non-neuronal Tdc1-GAL4 driver (Figure 6). In contrast, Tßh expression in subsets of OA/TA-neurons, using either Tdc2- or NP7088-GAL4 did not significantly affect the mutants' response (Figure 6), in contrast to a previous report [NP7088-Gal4, (Scheiner et al., 2014)]. These last two results also show that the UAS construct alone is not sufficient to bring a rescue. These results indicate that Tßh expression induced in neurons in the central nervous system or in non-neuronal cells, respectively, is sufficient to enhance the sugar responsiveness of *tßh*^*nM18*^ mutant flies.

### OA/TA-receptor manipulations on survival and sugar responsiveness

**Figure 7:**
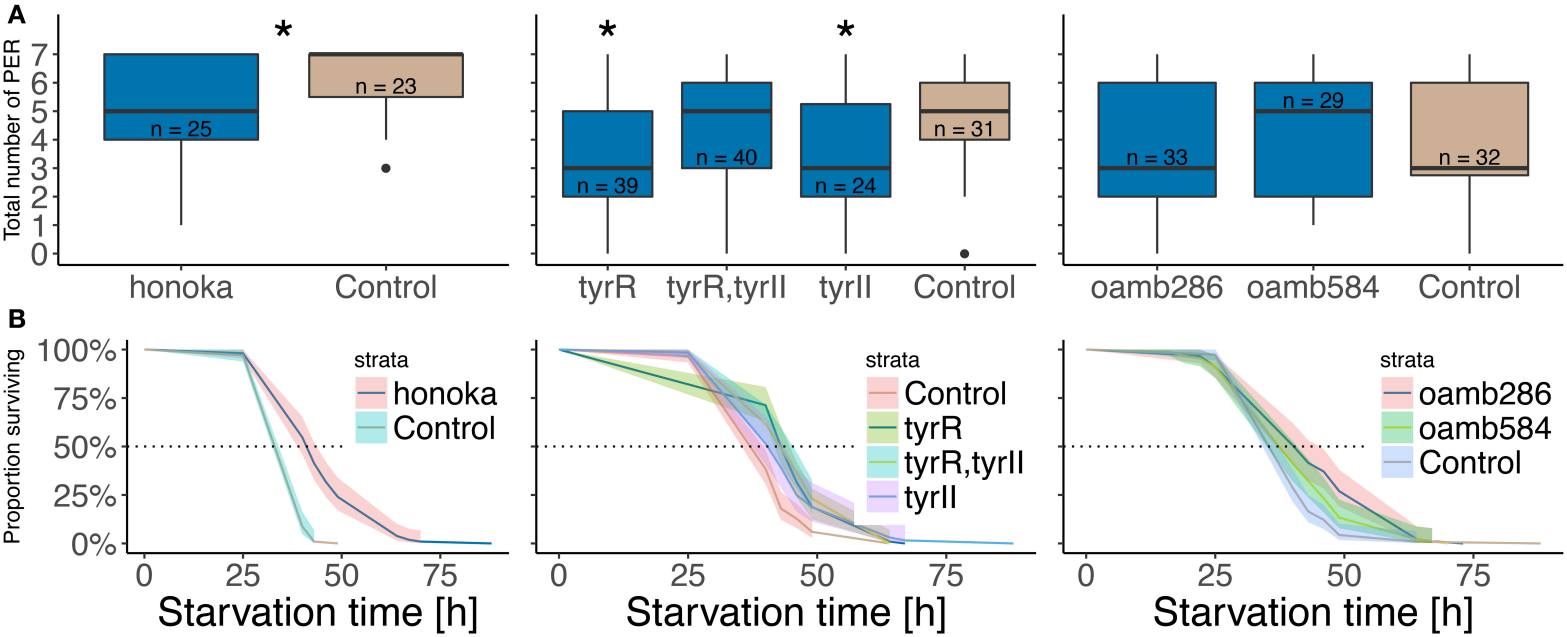
Starvation resistance and sugar responsiveness in TA- and OA-receptor mutants. Total number of proboscis extensions **(A)** and Kaplan Meier survival curve (**B**, see Figure 3) in different female mutants and their respective control strains. See text for fly strain labels. The three different groups were tested independently and are therefore statistically treated as different experiments. **(A)** Numbers indicate sample sizes, asterisks denote significant difference between the mutant and its respective control group (Wilcoxon rank sum test, *honoka p* = 0.0117, T*yr p* = 0.002911 and 0.007432). **(B)** 5 to 8 experiments were run per genotype, with about 35 flies per vial. Cox proportional hazards regression model was used to test statistical differences between mutants and their control, (different: *honoka p* = 1.4 × 10^−14^, *TyrR,TyrII p* = 10^−5^, *TyrII p* = 2.1 × 10^−6^, *oamb*^*286*^ *p* = 0.00029, not different: *TyR p* = 0.067, *oamb*^*584*^ *p* = 0.069, before bonferroni correction).

Because the *tßh* mutation leads to increased TA and decreased OA levels (Monastirioti et al., 1996), we performed additional experiments to disentangle the relative importance of each amine in the regulation of survival and sugar response. We tested mutants for several OA- and TA-receptors in our PER and survival under starvation condition assays (Figure 7, Table 1).

**Table 1:** Summary of TA- and OA-receptor mutant phenotypes.

		**Survival**	**Sugar response**
**TA-receptors**	*TyrR*^**f*05682*^	↑	↓
	*TyrRII^*Δ29*^*	—	↓
	*TyrRII-TyrR^*Δ124*^*	↑	—
	*honoka*	↑	↓
**OA-receptors**	*oamb*^**286**^	↑	—
	*oamb*^*584*^	—	—

*Horizontal lines indicate no effect. Arrows indicate significant difference to respective control and illustrate the trend of the data*.

The two TA-receptor mutants *TyrR*^*f05682*^ and *honoka* showed a decreased sugar response and an increased survival comparable to *tßh*^*nM18*^ mutants. In contrast, the double mutant *TyrII-TyrR*^*Δ124*^ showed an increase in survival but a normal sugar response, while *TyrRII*^*Δ29*^ shows normal survival but a decrease in sugar response. Finally, *oamb*^*286*^ mutants lived longer than their control, in contrast to a previously published report (Schwaerzel et al., 2003; Erion et al., 2012), while the *oamb*^*584*^ allele showed no phenotype. The receptor mutant data suggest that flies can exhibit a wild-type survival simultaneously with a lower sugar response (*TyrRII*^*Δ29*^), or a higher survival simultaneously with a wild-type sugar response (*oamb*^*286*^, double mutant *TyrII-TyrR*^*Δ124*^), suggesting that starvation affects sugar responsiveness and survival via different but amine-dependent pathways.

## Discussion

We have used genetic alterations of OA and TA action to elucidate the role of these amines in survival and sugar responsiveness of fruit flies. Our data suggest complex, central and peripheral actions of these amines on physiology and behavior.

We have shown that the *tßh* gene is involved in starvation-induced survival and an increase in sugar response. The phenotype was reported in females (Figure 1) and males (Figure 6), in three different genetic backgrounds (w+ and w−,*tßh*^*nM18*^; *hs-tßh* and w−,*tßh*^*nM18*^, U*AS-tßh*) and is independent of the egg-retention phenotype (Partridge et al., 1987), which is rescued in w−;*tßh*^*nM18*^;*UAS-tßh* control mutant flies (Figure 6). It is interesting to see that the sugar response phenotype appears to vanish with longer starvation periods (Yang et al., 2015). The phenotype was not found in previous reports focused on the learning phenotype of these flies (Schwaerzel et al., 2003), possibly because the assay used was dependent on locomotion, which is also affected in *tßh*^*nM18*^ mutants (Saraswati et al., 2004; Fox, 2006; Koon et al., 2010). Complementary results were obtained using a different approach in *Drosophila* (Scheiner et al., 2014) and also in *Apis mellifera* (companion paper).

### OA/TA and starvation resistance

Since sugar response is dependent on starvation (Colomb et al., 2009), a decreased sugar response as found in *tßh*^*nM18*^ mutants can be understood as resistance to the starvation treatment, an hypothesis that our results appeared to confirm. Indeed, we found that the levels of carbohydrates in the hemolymph of *tßh*^*nM18*^ mutant flies are higher after starvation than in control flies (Figure 2). Since trehalose constitutes the energy store of a fly and its hemolymph concentration reflects starvation level (Thompson, 2003; Isabel, 2004), it is reasonable to argue that the mutant flies were affected less by the starvation treatment than the controls, even though they were deprived of food for the same amount of time. This interpretation is also supported by longer survival of *tßh*^*nM18*^ mutants under starvation conditions [Figure 3, a result which was independently replicated (Scheiner et al., 2014; Li et al., 2016): our experiments were carried out before the ones cited]. Complementing our analysis in flies, injection of the OA-receptor antagonist epinastine in honey bees also prolonged survival (companion paper). Taken together, these results suggest that the absence of OA-signaling saves the mutant animal's energy, making the animals less sensitive to starvation, a conclusion in line with previous reports on the role of OA in trigylceride (Woodring et al., 1989; Erion et al., 2012) and carbohydrate (Blau et al., 1994; Park and Keeley, 1998) metabolism. One potential explanation for the reduced energy use may be a reduced locomotor activity in the mutant flies. We have tested flies in Buridan's paradigm (Colomb et al., 2012) and found several alterations to the locomotor pattern of *tßh*^*nM18*^ mutant flies (Damrau et al. in preparation).

### OA/TA and sugar responsiveness

While *tßh* is affecting starvation resistance, we asked whether the gene could also have a role in the neuronal modifications caused by starvation signals. Our results separate the starvation resistance from the sugar responsiveness phenotype. The sugar responsiveness phenotype is partially rescued by acute *tßh* expression, while expression during the starvation period had no effect (Figure 4). This suggests that the decrease of carbohydrate levels is not the only *tßh*-dependent starvation-induced alteration that leads to a normal sugar response. Indeed, the sensitivity of the sugar-sensing neurons is affected by TA/OA imbalance (Figure 5), but only after starvation. Interestingly, the control w+ strain did not show the expected (Meunier et al., 2007; Nishimura et al., 2012) increase in sensitivity after starvation (Figure 5A), while more common wild-type strains showed the increase in the same experiment (Figure 5B). Since the w+ control flies did show an increase in their proboscis extension response to sugar (Figure 1), there must be a modulatory mechanism downstream of taste receptor activity. Taken together, these data suggest that in addition to the internal state that is altered by starvation, both sensory transduction and the likelihood to extend the proboscis to the same sensory information are modified by starvation.

### Where is the site of OA/TA-action?

In order to identify the cells contributing to starvation resistance and sugar responsiveness, we expressed Tßh in different cells inside or outside the nervous system in the mutant flies, using the UAS/Gal4 system (Figure 6). The expected effect of this manipulation is a production of OA and a decrease in the concentration of TA in the affected cells. Ubiquitous expression of Tßh with the actin-Gal4 driver does increase the PER of starved flies. The non-neuronal Tdc1-GAL4-driver drives expression in crop and hind gut tyraminergic cells (Cole et al., 2005; Chintapalli et al., 2007; Blumenthal, 2009), that normally do not produce OA, but only TA (Monastirioti et al., 1995). Ectopic production of OA in these cells rescues the sugar responsiveness phenotypes (Figure 6). Because ectopic OA would lack necessary receptors, we tentatively interpret this result as an effect of presumably reduced TA levels. However, the OA produced might also be released into the hemolymph and taken up by neurons, as is proposed to happen when feeding OA (Schwaerzel et al., 2003; Scheiner et al., 2014). Interestingly, pan-neuronal Tßh expression with nsyb-Gal4, but not expression with drivers specifically labeling OA/TA neurons (tdc2-Gal4 and NP7088-Gal4), rescues the phenotype. These results suggest that both neuronal and non-neuronal tissues are affecting the starvation-induced increase in sugar responsiveness (and that the two most commonly used OA/TA drivers remain suboptimal tools to study OA action).

### OA and TA specificity

The TßH enzyme converts TA into OA such that *tßh*^*nM18*^ mutants not only lack OA but also accumulate TA. To disentangle the roles of the two amines, we tested OA- or TA-receptor mutants in two experiments: starvation resistance and sugar responsiveness (Figure 7). Perhaps not surprisingly, given that several processes appear to mediate both starvation-induced effects, we found the sugar responsiveness and the starvation resistance phenotypes of the tested mutants to be separable: some mutants exhibit a phenotype in none (*oamb*^*584*^), both (*tßh*^*nM18*^, *honoka*), or in individual assays: only in starvation resistance (*oamb*^*286*^, *TyrII-TyrR*^*Δ124*^) or only in sugar responsiveness (*TyrR*^*Δ29*^). These results reinforce our previous conclusion that starvation resistance and sugar responsiveness are not mediated by the same OA/TA-cells and receptors, but by different sub-populations. In addition, the data indicate that both OA and TA play a role in starvation-induced sugar responsiveness. OA- and TA-receptor mutants tend to perform similarly, suggesting they may not be counteracting each other in this behavior, as previously suggested for crawling behavior (Saraswati et al., 2004) or for flight (Brembs et al., 2007).

## Conclusions

Taken together with the experiments from our accompanying paper (Buckemüller et al., 2017), our results suggest that the OA/TA-system is involved in both the physiological and the behavioral changes that follow starvation, and that these changes are regulated independently. They also show that the behavioral change is due not only to a modulation of the taste neuron activity and to action of TA-specific cells in peripheral, non-neuronal organs, but that a more central effect is probably at play. Finally, these data as well as others (in prep.) suggest that some aminergic pathways operate in a dose-dependent manner and are therefore difficult to dissect using standard transgenic or pharmacological rescue approaches.

## Author contributions

CD and JC: Design of experiments, collection and analysis of data, writing and editing of the manuscript. NT and TT: Performed electrophysiology experiments, analyzed data, edited manuscript. BB: Design of experiments, analysis of data, writing and editing of the manuscript.

### Conflict of interest statement

The authors declare that the research was conducted in the absence of any commercial or financial relationships that could be construed as a potential conflict of interest. The reviewer VM and handling Editor declared their shared affiliation, and the handling Editor states that the process nevertheless met the standards of a fair and objective review.
